# mTOR kinase inhibitor pp242 causes mitophagy terminated by apoptotic cell death in E1A-Ras transformed cells

**DOI:** 10.18632/oncotarget.6457

**Published:** 2015-12-04

**Authors:** Serguei A. Gordeev, Tatiana V. Bykova, Svetlana G. Zubova, Olga A. Bystrova, Marina G. Martynova, Valery A. Pospelov, Tatiana V. Pospelova

**Affiliations:** ^1^ Institute of Cytology, Russian Academy of Sciences, St. Petersburg, Russia; ^2^ Saint Petersburg State University, St. Petersburg, Russia

**Keywords:** mTORC1, rapamycin, pp242, autophagy, mitophagy

## Abstract

mTOR is a critical target for controlling cell cycle progression, senescence and cell death in mammalian cancer cells. Here we studied the role of mTOR-dependent autophagy in implementating the antiprolifrative effect of mTORC1-specific inhibitor rapamycin and ATP-competitive mTOR kinase inhibitor pp242. We carried out a comprehensive analysis of pp242- and rapamycin-induced autophagy in ERas tumor cells. Rapamycin exerts cytostatic effect on ERas tumor cells, thus causing a temporary and reversible cell cycle arrest, activation of non-selective autophagy not accompanied by cell death. The rapamycin-treated cells are able to continue proliferation after drug removal. The ATP-competitive mTORC1/mTORC2 kinase inhibitor pp242 is highly cytotoxic by suppressing the function of mTORC1-4EBP1 axis and mTORC1-dependent phosphorylation of mTORC1 target - ULK1-Ser757 (Atg1). In contrast to rapamycin, pp242 activates the selective autophagy targeting mitochondria (mitophagy). The pp242-induced mitophagy is accompanied by accumulation of LC3 and conversion of LC3-I form to LC3-II. However reduced degradation of p62/SQSTM indicates abnormal flux of autophagic process. According to transmission electron microscopy data, short-term pp242-treated ERas cells exhibit numerous heavily damaged mitochondria, which are included in single membrane-bound autophagic/autolysophagic vacuoles (mitophagy). Despite the lack of typical for apoptosis features, ERas-treated cells with induced mitophagy revealed the activation of caspase 3, 9 and nucleosomal DNA fragmentation. Thus, pp242 activates autophagy with suppressed later stages, leading to impaired recycling and accumulation of dysfunctional mitochondria and cell death. Better understanding of how autophagy determines the fate of a cell - survival or cell death, can help to development of new strategy for cancer therapy.

## INTRODUCTION

The mammalian target of rapamycin (mTOR) is a serine/threonine kinase that integrates a multiple of extracellular and intracellular signals to drive cellular growth, proliferation, autophagy or senescence [1–6]. Mammalian Target of Rapamycin Complex 1 (mTORC1) promotes cell growth by inducing anabolic and inhibiting catabolic processes [7]. Numerous observations support the importance of mTOR pathway in cancer development, particularly oncogenic activation of mTOR signaling induces processes required for cancer cell proliferation [8]. mTORC1 is a positive regulator of protein and lipid synthesis [9–12] as well as energy metabolism and general negative regulator of autophagy and lysosome biogenesis [13, 14]. Anticancer drugs inhibiting the activity of mTORC1 and mTORC2 complexes potentially can suppress cancer cell proliferation. In accordance with this, an allosteric mTORC1 inhibitor rapamycin and rapalogs activate a wide spectrum of responses [3, 15–17]. However, although rapamycin inhibits the proliferation of certain cancer cell lines *in vitro*, it is not effective in a large number of human malignancies *in vivo* [18–23]. In contrast, inhibitors of kinase mTOR domain is more effective in inhibiting proliferation of cancer cells and have more pronounced antiproliferative effect on tumor *in vivo* [24–28] due to suppression of both mTORC1 и mTORC2 complexes [29].

Autophagy is an important cellular mechanism responsible for degradation of dysfunctional cellular organelles and proteins in all living cells, mediat­ing the removal of damaged organelles and proteins, which are digested and recycled for cellular needs again [30]. Autophagy, also known as a reason of programmed cell death type II (autophagic death), represents an alternative tumor-suppressing mechanism [31]. Unlike apoptosis, which is a caspase-dependent process characterized by chromatin condensation, nuclear shrinkage and DNA fragmentation without major structural changes in cytoplasm, autophagy is a caspase-independent process characterized by the accumulation of autophagic vacuoles in the cytoplasm connected with degradation of proteins, mitochondria, ribosomes and the endoplasmic reticulum, which precedes the destruction of the nucleus. In connection with these, autophagy may be important in determining the response of cancer cells to anticancer therapy, especially in the case of apoptotic resistance of many cancers to radio- and chemotherapy [32, 33].

In this paper, we focused on the study of antiproliferative effect of mTORC1 inhibitor rapamycin and an inhibitor of the mTOR kinase domain pp242 on tumor rodent E1A + cHa-Ras (ERas) cells. In particular, we checked how the mTOR inhibitor-induced autophagy can be involved in suppression of proliferation by triggering cell death. We showed that rapamycin induced in ERas cells the process of non-selective autophagy, whereas pp242 induced selective autophagy. Suppression of proliferation by mTOR kinase inhibitor pp242 is due to induction of a specific form of autophagy - mitophagy that eventually causes the cell death. By using immunofluorescence, Western blot and electron microscopy analyses, we checked mTORC1-4EBP1 and mTORC1-S6 axes inhibition, ULK1,2 phosphorylation and activation of autophagy markers - LC3, p62/SQSTM and Beclin1 after short-term and long-term treatment of ERas cells with the inhibitors. Antiproliferative effect of mTOR inhibitor pp242 is closely connected with strong inhibition mTORC1-4EBP1 axis, mTORC1-dependent suppression of ULK1,2-Ser757 phosphorylation, LC3-II accumulation and a decrease of Beclin1 expression. According transmission electron microscopy (TEM) data, ERas cells shortly treated with pp242 showed numerous severely damaged mitochondria characterized by an intense vacuolization and destruction of mitochondrial cristae. Furthermore, the accumulation of single membrane-bound autophagic vacuoles, containing mitochondria (mitophagy) results in the cell death. Despite the lack of typical picture of apoptotic death (chromatin condensation, apoptotic body formation, cytoplasmic blebbing), the ERas-treated cells undergoing mitophagy revealed both caspase-3, 9 activation and nucleosomal DNA fragmentation ladder.

## RESULTS

### PP242 but not rapamycin irreversibly inhibits proliferation of ERas-transformed cells

**Figure 1:**
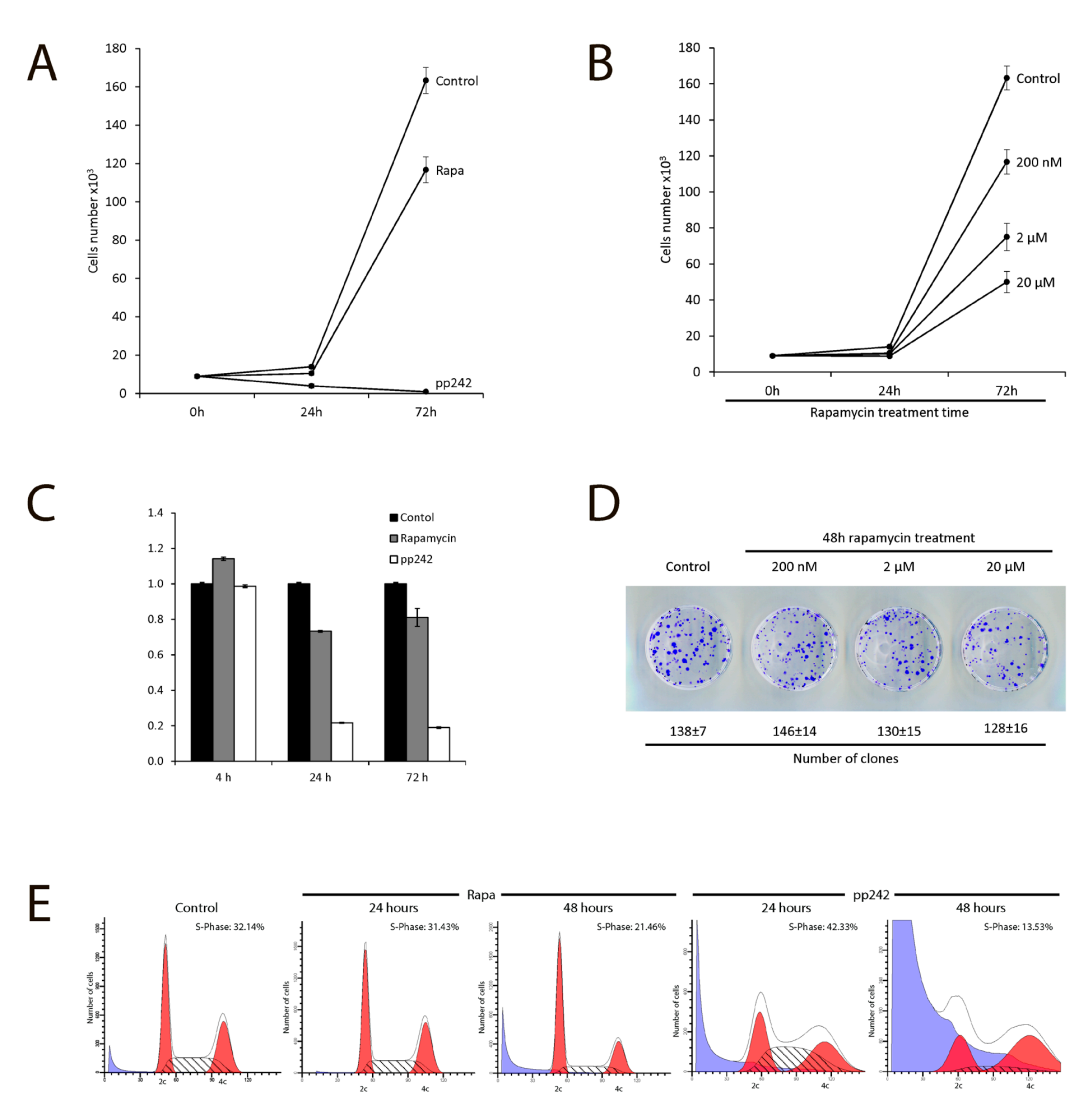
Antiproliferative activity of mTOR inhibitors rapamycin and pp242 **A.** Growth curves of untreated and treated ER cells. Cells seeded at initial density of 3 × 10^4^ per 30-mm dish, then were treated with the mTOR inhibitors, the number of cells was counted after 24 h and 72 h of the treatment. Data are presented as mean ±S.E.M. of three independent replicates (*n* = 3). Rapamycin (Rapa) - concentration 200nM, pp242 concentration 1500 nM. **B.** Growth curves of Eras cells treated with different concentrations of rapamycin. Experimental conditions as in Figure A. **C.** MTT test for viability of Eras cells treated with rapamycin and pp242. The cells were plated on 96-well plates at the initial density of 2500 cells/well in triplicates. After 24 h of growth, the cells were treated with the drugs and then were processed with MTT reagent for further measurement at 570 nM. Data are presented as mean ±S.E.M. of three independent replicates (*n* = 3). **D.** Clonogenic survival of ERas cells treated with for 48 h with the indicated concentrations of rapamycin. Cells were treated with different concentration of Rapa for 48 h, after that were seeded at 200 cells per 30 mm dish in drug free medium. Clones were stained after 5 days. Data are presented as mean ±S.E.M. of three independent replicates (*n* = 3). **E.** Cell cycle distribution of Eras cells treated with Rapa (200nM) and pp242 (1500 nM) for 24 h and 48 h by flow cytometry of propidium iodide-stained cells. The percentage of S-phase cells is indicated.

Firstly, we assessed a suppression effect of pp242 and rapamycin on the proliferation of ERas-transformed cells. Rapamycin was used as a very specific allosteric inhibitor of mTORC1, while pp242 has been shown to suppress the activity of both TORC1 and TORC2 complexes [18–21]. According to the growth curves data presented in Figure 1A, pp242 completely suppressed proliferation after 48 h treatment at concentration 1500 nM, whereas 200 nM Rapa inhibited only by 30%. Moreover, rapamycin was unable completely suppress proliferation even at the concentration 20 000 nM (Figure 1B). Similar conclusion follows from MTT assay (Figure 1C). So, after 24 h treatment with pp242, MTT test reveals a sharp drop in cell viability (up to 90%) that is characteristic for dying cells. In contrast, according to MTT assay rapamycin decreases the viability after 24 h treatment, but the viability restores to 72 h. According to the clonogenic survival test of rapamycin at low or very high dose (20 000 nM) does not cause cell death (Figure 1D). This means that the temporary inhibition of proliferation by Rapa may be associated with resistance of ERas cells to rapamycin. The flow cytometry data show an accumulation of ERas cells in G1-phase after rapamycin treatment and the appearance of the sub-diploid peak in pp242 - treated cells, which is a characteristic of cells undergoing apoptosis. (Figure 1E).

**Figure 2:**
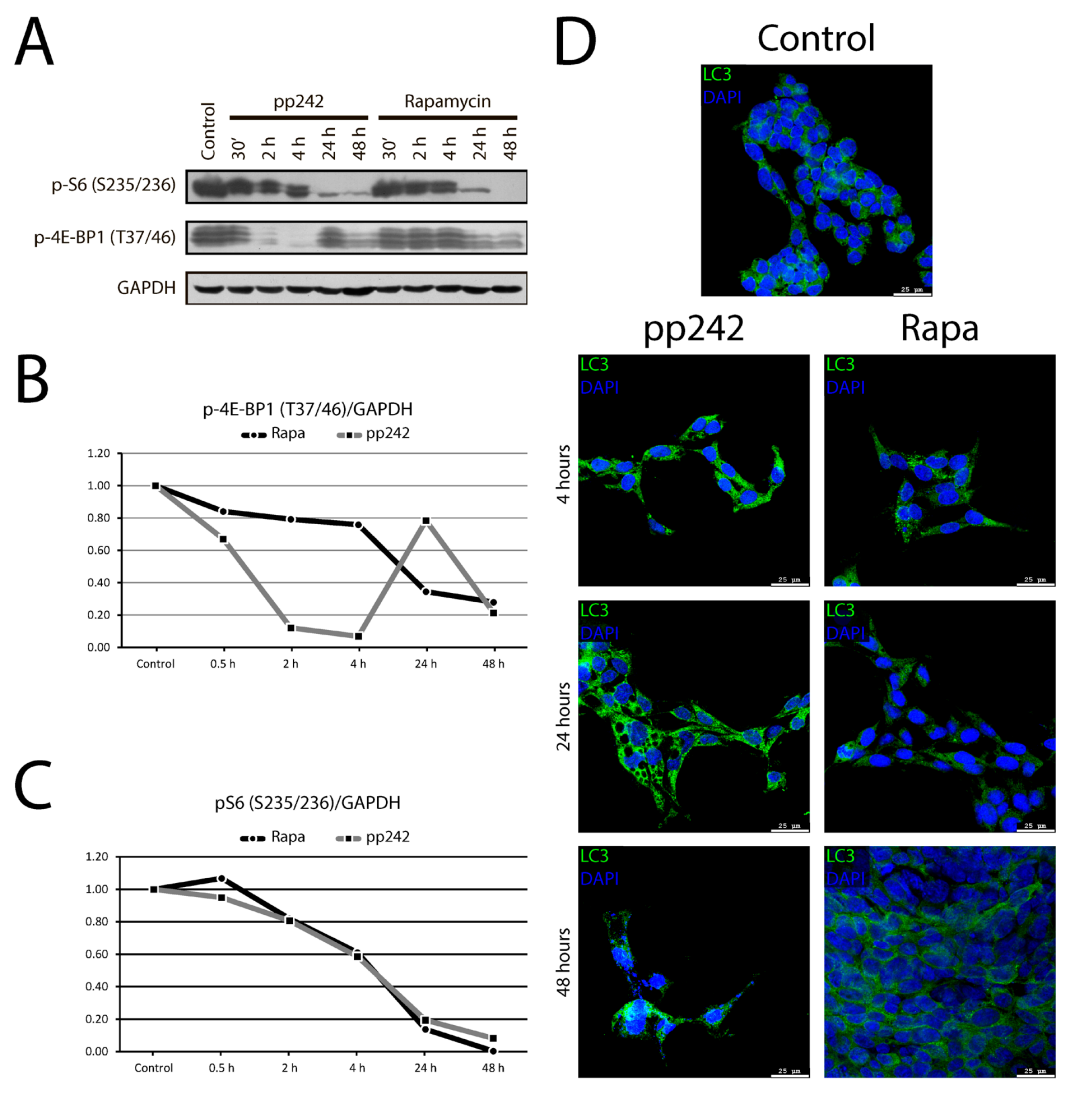
The dynamics of phosphorylation of mTORC1 targets and LC3 accumulation in Eras cells treated with mTOR inhibitors **A.** Western blot analysis of short-term (30 min, 2 h, 4 h) and long-term (24 h and 48 h) effects of Rapa and pp242 on phosphorylation of S6 and 4EBP1. ERas cells were treated with Rapa or pp242 in the indicated times and lysed. Rapa was used at 200 nM, pp242 at 1500 nM. Immunoblotting was performed with antibodies to pS6-Ser235/236 and p4EBP1-Thr37/46. **B.** Densitometry of bands intensity 4EBP1-Thr37/46 from Figure 2A by using GelPro software. **C.** Densitometry of bands intensity pS6-Ser235/236 from Figure 2A by using GelPro software. **D.** Immunofluorescence of LC3 protein in control Eras cells and treated with mTOR inhibitors: Rapa (200nM) and pp242 (1500 nM). Cells were treated with mTOR inhibitors for 48 hours and stained with antibody to a marker of autophagy LC3 (PM036, MBL).

**Figure 3:**
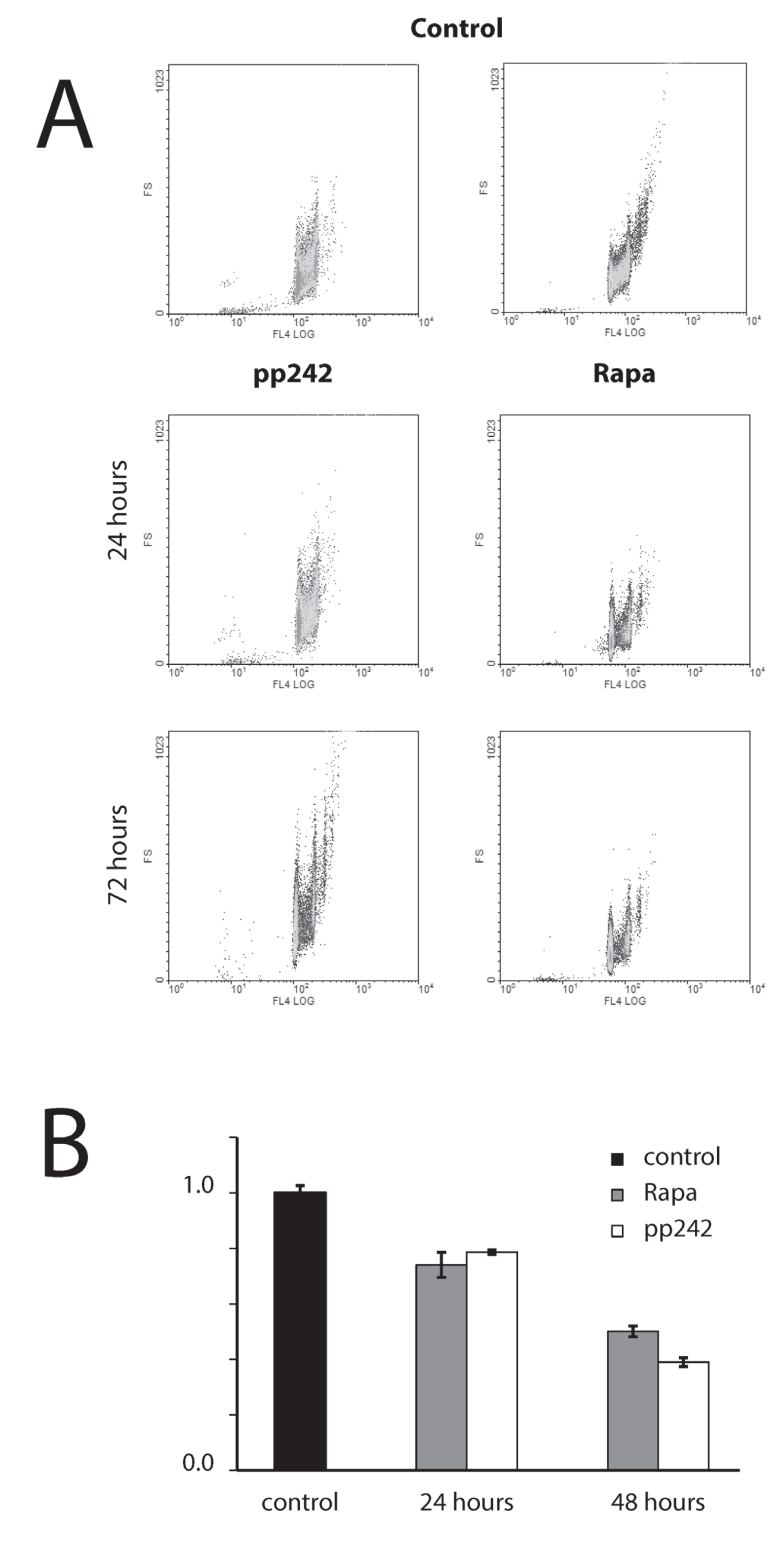
Analysis of the cell size and the protein content of ERas cells treated with rapamycin and pp242 **A.** Cells were treated with 200 nM of rapamycin and 1500 nM of pp242 for 24 h and 48 h. The size of control and inhibitors-treated cells was estimated by means of cytometric light scattering of propidium iodide-stained cells. **B.** Analysis of the protein content per cells as assessed by the Bradford's method.

The mTORC1 pathway regulates protein synthesis and translation by phosphorylation of 70S6 kinase (S6K) at Thr389 and eIF4E-binding-protein 4EBP1 at Thr37/46. Western blot data show that rapamycin and pp242 have different effects on mTORC1-4EBP1 and mTORC1-S6 axes. We compared the short-term (30 min, 2 h, and 4 h) and long-term (24 h and 48 h) effects of rapamycin and pp242 on phosphorylation of S6 and 4EBP1 (Figure 2A). Thus, pp242 decreases the phosphorylation of 4EBP1 at Thr37/46 up to about 40% after 30 min treatment and completely inhibits their phosphorylation after treatment for 2 h and 4 h (Figure 2B), while inhibitory effect of rapamycin is much less effective after short-term treatment. Only 24 h rapamycin treatment results in suppression of 4EBP1 phosphorylation. Rapa and pp242 inhibited phosphorylation of S6 protein with a similar efficacy by about 40% after 4 h, but after 24 h the inhibition was 80-90% of control values (Figure 2C). In contrast to pp242, Rapa decreases not only the protein content per cell, but also reduces the cell size (Figure 3A, 3B).

**Figure 4:**
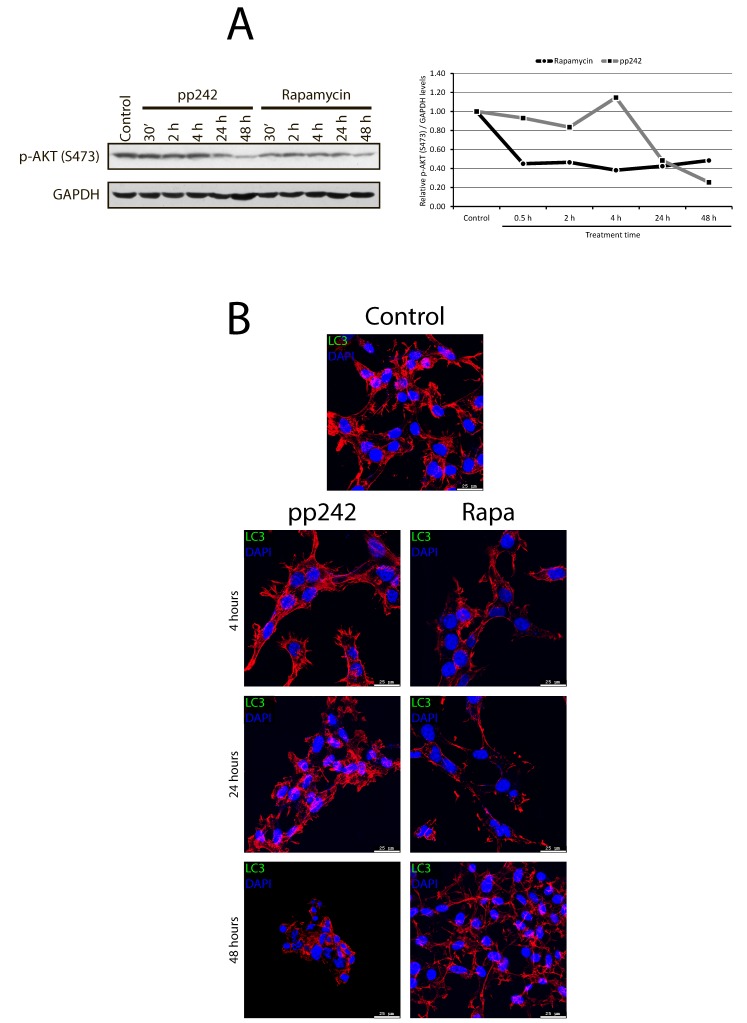
The dynamics of mTORC2 activity assessed by phosphorylation of its target PKB/Akt-S473 **A.** Western blot analysis of lysate from Eras cells treated with rapamycin or pp242 at various time intervals. Rapamycin was used at 200 nM, pp242 at 1500 nM. Blots were stained with antibodies to PKB/Akt-Ser473 (left panel). Right panel shows densitometry of PKB/Akt-S473 band intensity from Figure 4A by using GelPro software. **B.** mTOR kinase inhibitor pp242 suppresses ERas proliferation without affecting the actin stress fibers organization. Cells were stained for actin with Rhodamin-Falloidin (red) and for DNA with DAPI (blue). The images were taken with the confocal microscope (Leica). The images are representative of more than 100 cells for each inhibitor.

Incomplete inhibition of 4EBP1 phosphorylation caused by Rapa as compared with pp242 after short-term treatment can be due to the involvement of mTORC2/PKB/Akt in the regulation of phosphorylation of 4EBP1 [34]. To analyze this possibility, we checked the effect of pp242 and rapamycin on the phosphorylation of PKB/Akt-Ser473, which is a key mTORC2 target. Data presented in Figure 4A show that both pp242 and rapamycin inhibit PKB/Akt-Ser473 phosphorylation but with different efficiency. Surprisingly, PKB/Akt-Ser473 phosphorylation has been inhibited after short-term rapamycin treatment to a greater extent than after pp242. The more complete inhibition of PkB/Akt-Ser473 by Rapa than by pp242 after short-term treatment indicates that the activity of mTORC2 is not the cause of a higher level of 4EBP1 phosphorylation as downstream mTORC2/PKB/Akt target in Rapa-treated cells.

It is known that mTORC2 can be involved in the actin cytoskeleton rearrangement, influencing the formation of actin stress fibers [35, 36]. We examined the morphology of actin stress fibers in ERas cells treated with pp242 and rapamycin (Figure 4B). After pp242 and rapamycin treatment for 4 h, 12 h and 24 h no obvious effect on the morphology or abundance of actin stress fibers was found. Thus, mTOR kinase inhibitor pp242 suppresses ERas proliferation without affecting the actin stress fibers organization.

### Suppression of mTOR activity by rapamycin induces the non-selective autophagy, whereas pp242 activates mitophagy in ERas transformed cells

**Figure 5:**
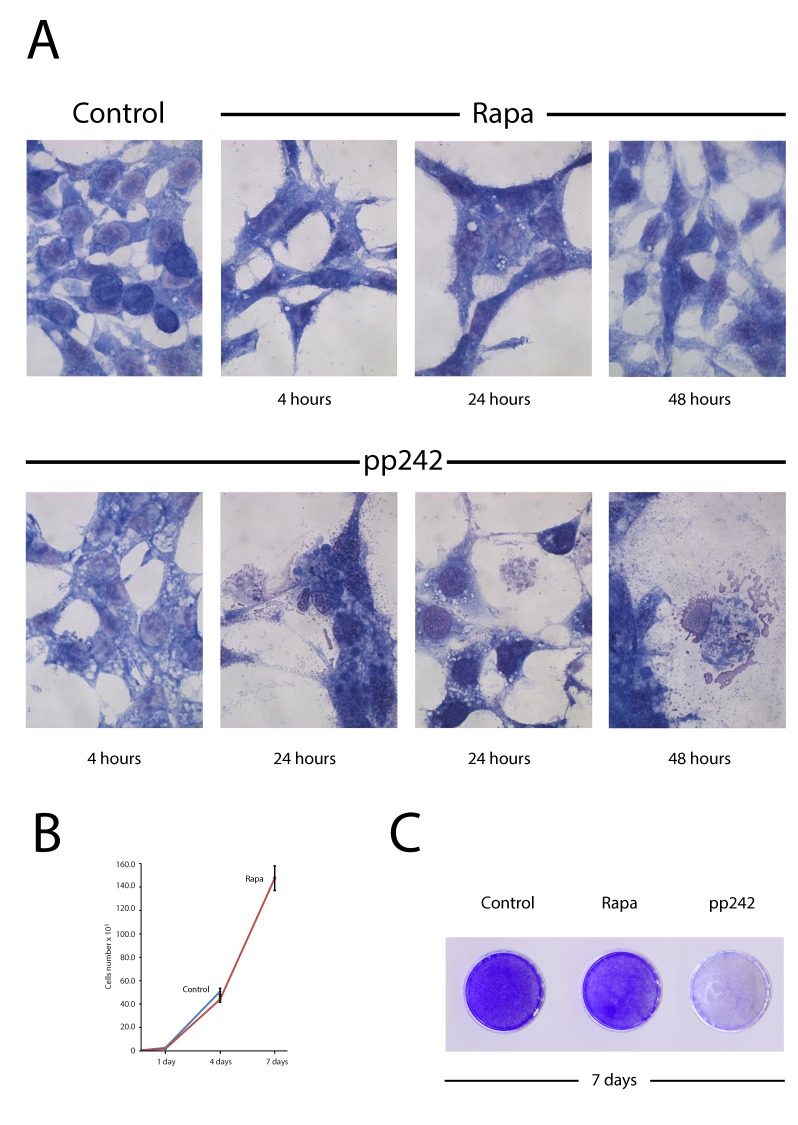
Rapamycin-induced autophagy is not accompanied by destructive changes in the cytoplasm of ERas cells and does not lead to irreversible inhibition of proliferation **A.** Morphology of ERas cells treated with rapamycin and pp242 for 4 h, 24 h and 48 h. Rapa concentration was 200 nM and pp242 -1500 nM. Rapa causes the formation of autophagic vacuoles visible to the eye that is not accompanied by a destruction of the cytoplasm structure, while pp242 induces the formation of giant vacuoles and a destruction of the cytoplasm. Images were acquired in transmitted light, magnification 40× 10. **B.** Rapamycin-treated cells retain the ability to proliferate. Growth curves of the rapamycin-treated cells (200 nM). Cells overcome the anti-proliferative effect of rapamycin after 7 days of cultivation without washing the inhibitor. **C.** A picture of cultured plates stained with crystal violet after 7days of rapamycin or pp242 treatment.

The mTORC1 takes a part in regulation of cell proliferation through the ribosome biogenesis and translation initiation as well as it controls the catabolic processes being one of the key regulators of the autophagy [37–39] and cellular senescence [40, 41]. Data presented in Figure 5A show the morphology of cells after treatment with pp242 and rapamycin. The kinase inhibitor causes rapid formation of cytoplasmic vesicles, the number and size of which increase on as treatment of the culture with an inhibitor continues (Figure 5A). At the same time, after 24 h treatment there can be seen the destructive changes in the cytoplasm: the appearance of enormous size empty cisterns, which shift the nucleus to the periphery of the cytoplasm, and a sharp drop in cell density. After 48 h, there are only sporadic, much flattened large cells containing large vacuoles with no apparent membrane. In contrast, in rapamycin-treated cells there are only sporadic vacuoles within the cells and no visible destruction of the cell morphology. The long-term treatment with pp242 for 7 days causes the total death of cell population - no single colony can be seen after the prolonged pp242 treatment (Figure 5C). However, rapamycin-treated cells, after a temporary suppression of proliferation, start to divide again with high efficiency, eventually forming a cell monolayer with a similar density as in control (Figure 5B, 5C).

**Figure 6:**
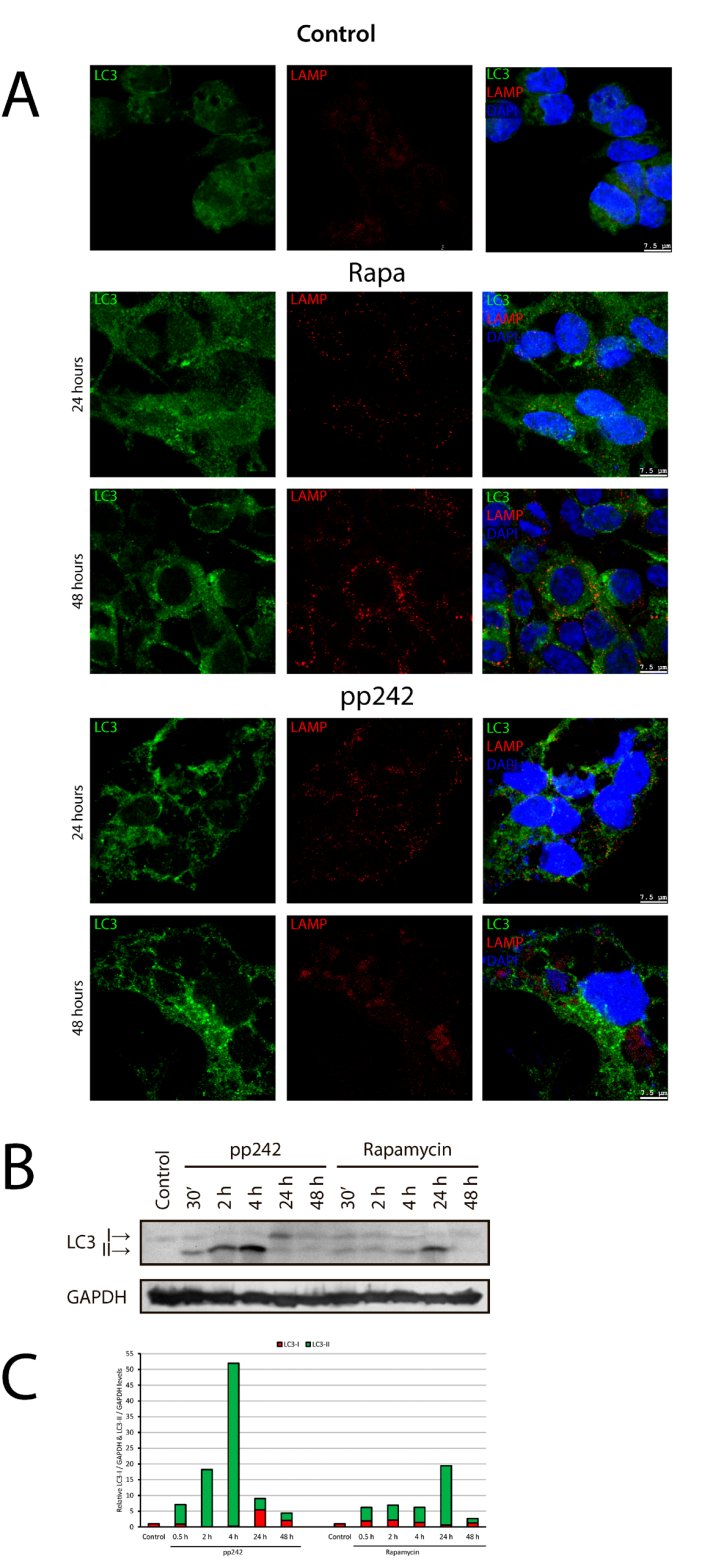
Immunofluorescent and Western blot analysis of rapamycin- and pp242-induced autophagy **A.** The kinetics of LC3 and LAMP1 accumulation at 2 and 4 hours after rapamycin and pp242 treatment. Cells were stained with antibodies to LC3 (green) and LAMP1 (red). DNA was co-stained with DAPI (blue). **B.** Western blot for conversion of LC3-I (cytosolic form) to LC3-II (membrane-bound) form in ERas cells treated with Rapa and pp242. **C.** Graphical representation of the blot data by means of densitometry of the LC3-I (green) and LC3-II (red) forms.

To study the activation of autophagy in ERas cell after treatment with mTOR inhibitors, we used immunofluorescence staining with antibodies against LC3 (Atg8) protein, which is a key marker of autophagy. These data indicate that control ERas cells have a basal level of autophagy (Figure 6A) likely protecting the viability of the Ras-expressing cells [42, 43]. The rapid accumulation LC3 in rapamycin- and pp242-treated cells has been detected after 4 h, but after 24 h of treatment with rapamycin there is a significant decrease of LC3 staining, while in the pp242-treated cells the LC3 signal remains high (Figure 6A). Comparison of immunofluorescence and Western blot data for LC3 staining (Figure 6B) shows that pp242 causes both accumulation of LC3 and intensive conversion of LC3-I cytoplasmic form to autophagosome membrane-associated form LC3-II, thereby reflecting the formation of autophagosomes. Treatment with pp242 for 4 h leads to a complete conversion of LC3-I to LC3-II, suggesting a rapid and effective activation of autophagic process. However, after prolonged pp242 treatment the overall content of LC3 falls, the proportion of LC3-II form decreases and the ratio of LC3-I to LC3-II changes in such a way that characterizes the reduction of autophagy. Nevertheless, even 24 h and 48 h after exposure with pp242 the LC3 signal still exists (Figure 2D), indicating the low level of autophagic activity and the incompleteness of its full cycle. Rapa caused a slight accumulation of the overall LC3 staining (IF) and a low level of LC3-I to LC3-II conversion, more noticeable after 24 h (Figure 6B). After 48 h of Rapa treatment process of autophagy is comparable to control and it does not affect the process of proliferation.

**Figure 7:**
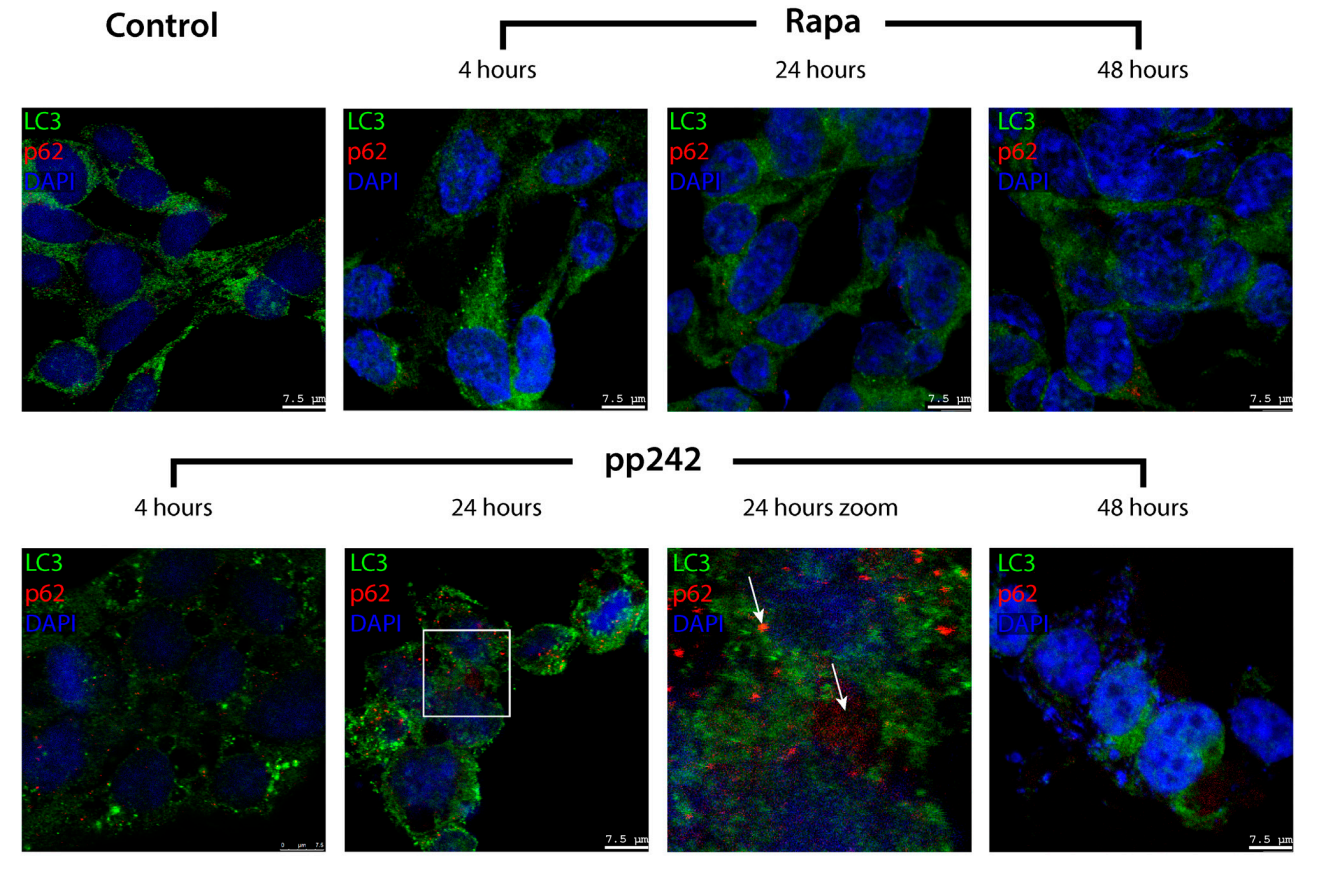
Degradation of autophagy-specific substrate p62/SQSTM and autophagy marker LC3 is disturbed in pp242-treated ERas cells Cells were plated on glass coverslips, treated with inhibitors, fixed and then stained with antibodies to LC3 (green) and p62/SQSTM (red). The arrows indicate the areas of p62/SQSTM accumulation in autophagolysosomes as an evidence for a suppression of the later stages of autophagy. A section indicated by the square is shown at higher magnification in the panel 24 h Zoom. Confocal images are shown.

Another important marker of autophagy is p62/SQSTM protein, which is involved in the flux of autophagy by binding to LC3 and then it degrades [44–46]. Thus, the expression level of p62/SQSTM in the cell is inversely correlated with the autophagic activity [47, 48]. According to immunofluorescence data of p62/SQSTM staining, the process of autophagy induced by Rapa proceeds with a normal activity, whereas pp242-induced autophagy is not accompanied by degradation of p62/SQSTM protein, which is present until the last time point of pp242 treatment (Figure 7), indicating severe failure of late stages of autophagic flux.

**Figure 8:**
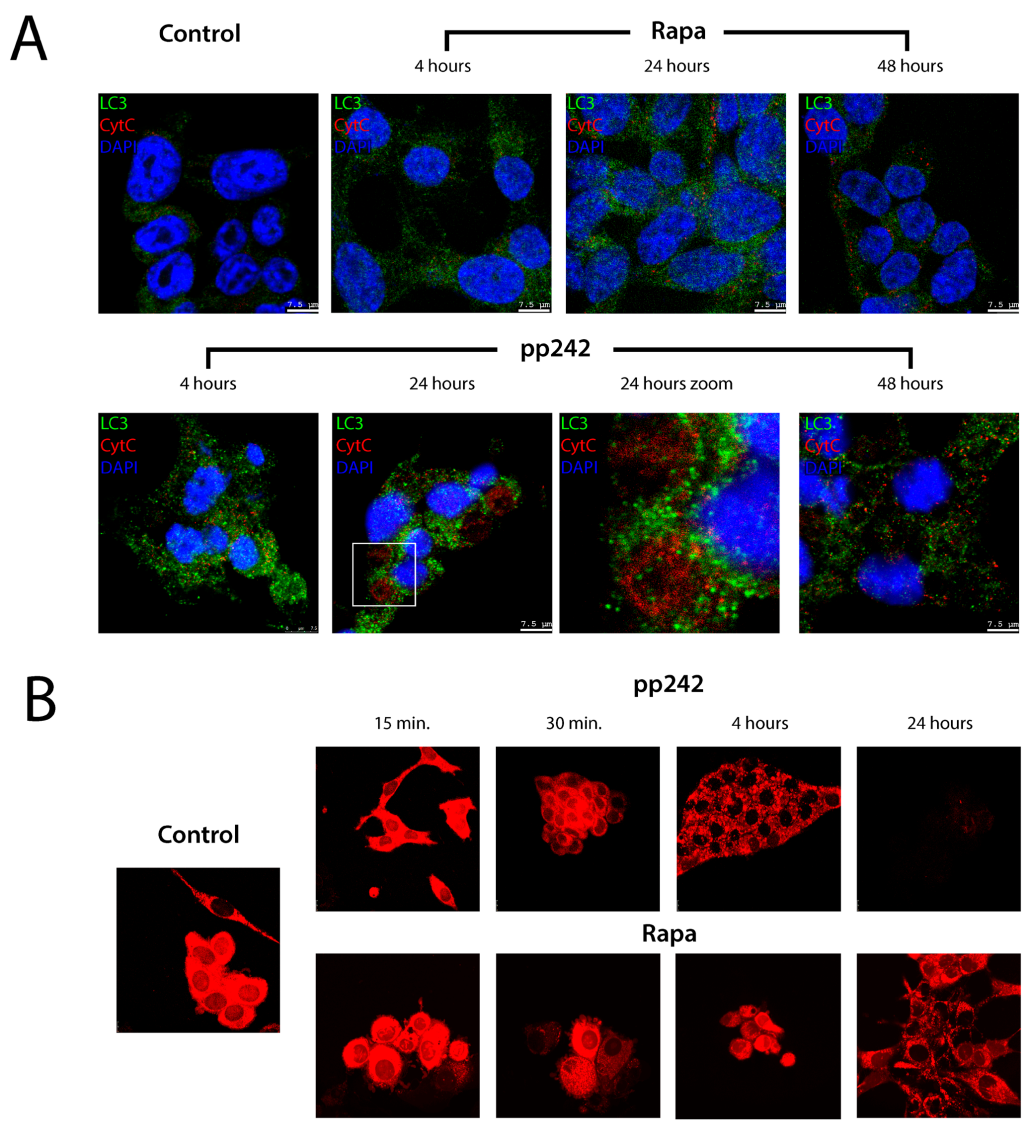
The intracellular distribution of the mitochondrial cytochrome C and mitochondrial activity after exposure of ERas cells with mTOR inhibitors **A.** Cytochrome C staining in ERas cells treated at different time intervals with rapamycin and pp242. Cells were plated on glass coverslips, treated with inhibitors, fixed and then stained with antibodies to LC3 (green) and cytochrome C (red). DNA staining with DAPI (blue). The arrows indicate the cytochrome C in giant vacuoles of pp242-treated cells for 24 h. A section indicated by the square is shown at higher magnification in the panel 24 h Zoom. After 48h cytochrome C staining completely disappears, while LC3 signal is detected even after 48 h. In rapamycin-treated cells the free cytochrome C is not detected at all stages of treatment. The images were taken with the confocal microscope (Leica). **B.** Mitochondrial activity assessed by an *in vivo* staining dye TMRM. In pp242-treated cells TMRM staining is clustering (30 min and 4 h), then by 24 h color disappears.

Autophagy induced by mTORC1 and mTORC2 inhibitors has very important distinctions: while rapamycin induced a non-selective autophagy, pp242 triggered in ERas cells is a selective autophagy, namely mitophagy. It is known that mitophagy is the first response of cells to different types of cell stress and damages [49]. To prove that the pp242-induced autophagy can be classified as mitophagy, we performed a series of experiments for analyses of mitochondrial activity. In particular, we used antibody to cytochrome C (cyt C) to assess the localization and dynamics of the mitochondrial cyt C, *in vivo* mitochondria-specific TMRM dye and the transmission electron microscopy (TEM) of mitochondria structure. Immunofluorescent analysis of cyt C distribution and its co-localization with LC3 within the autophagosomes showed that on early steps there is an accumulation of mitochondrial cyt C in the autophagosome cavities, but after 48 h of pp242 treatment cyt C is no longer detected in the autophagosome vesicles as well as LC3 (Figure 8A). However, the LC3 staining still present in the cytoplasm at this time indicating on incompleteness of autophagic cycle even after complete degradation of damaged mitochondria as shown by the absence of the cyt C signal. The distribution of cyt C in rapamycin-treated cells evidences in favor of preserving the integrity of mitochondria. According to the intensity of the TMRM staining used to assess the functional state of mitochondria, Rapa does not noticeably change its intensity and intracellular distribution. But pp242 firstly causes the TMRM clustering and a decrease in the intensity of luminescence but after 24 h-treatment its luminescence quenches (Figure 8B) that can be considered as a serious damage of mitochondrial functions induced by pp242.

### Transmission electron microscopy of mTOR inhibitor-treated ERas cells

**Figure 9:**
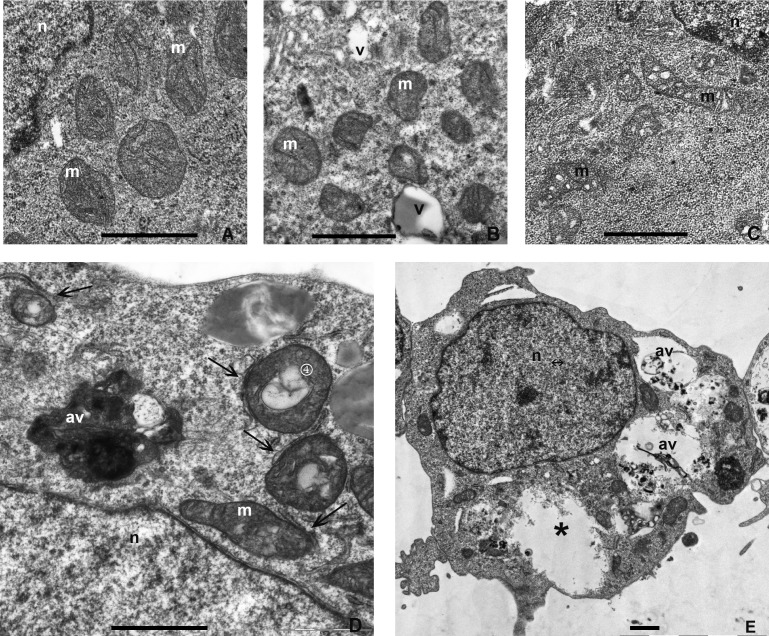
Transmission electron microscopy (TEM) images showing the ultrastructure of control or treated with rapamycin and pp242 ERas cells **A.** The cytoplasm of untreated cells (control) to demonstrate the normal ultrastructure. Mitochondria have well-ordered densely packed cristae. **B.**, **C.** TEM images for ERas cells at 2.5 h B. and 24 h C. after rapamycin treatment. The ultrastructural changes in the rapamycin-treated cells at both time points represent slight alterations of mitochondria as mild vacuolization and disordering of cristae. **D.**, **E.** TEM images for ERas cells at 4 h D. and 24 h E. after pp242 treatment. D. At 4 h, cells forms autophagic vacuoles containing destroyed mitochondria encircled by a single membrane, mitochondria demonstrating an intense vacuolization and cristae destruction. Note the close contacts between cisterns of the rough endoplasmic reticulum and mitochondria (arrows). **E.** At 24 h, cells exhibited significantly increased in size autophagic vacuoles containing presumably the remains of severely digested mitochondria. Empty membranes lacking the cavities (asterix) were also observed within the cytoplasm. Designations: av, autophagic vacuole; m, mitochondria; n, nucleus; v, vacuole. Scale bars corresponds to 1 μm.

To evaluate the fine structure of mitochondria in ERas cells treated with mTOR inhibitors rapamycin and pp242, we used the transmission electron microscopy (TEM). Control cells revealed the presence of microvilli on the cell surfaces, central or exocentric lobulated nucleus, free ribosome-rich cytoplasm, non-prominent Golgi apparatus, smooth endoplasmic reticulum, a number of small vacuoles, and rounded or oval mitochondria with densely packed cristae (Figure 9A).

Rapamycin-treated ERas cells demonstrate no significant changes of cell ultrastructure after 4 h and 24 h of exposure to Rapa as compared with the controls. The only evident ultrastructural alteration is a mild vacuolization of mitochondria and less densely packed mitochondrial cristae (Figure 9B and 9C). The ERas cells treated with pp242 for 2.5 h showed the presence of numerous severely damaged mitochondria characterized by an intense vacuolization and destruction of mitochondrial cristae (Figure 9D). Furthermore, there observed accumulation of single membrane-bound autophagic vacuoles containing the organelle debris that appeared to be the remains of digested mitochondria. It is appropriate to note the emergence of numerous tight contacts between cisterns of the rough endoplasmic reticulum and mitochondria (Figure 9D). No significant distinctions found in other intracellular organelles. After 24 h, the cells exhibited accumulation of significantly extended autophagic vacuoles and large empty membrane-lacking cavities within the cytoplasm (Figure 9E).

### PP242-induced mitophagy is terminated by the cell death with DNA fragmentation pattern characteristic to apoptosis

The pp242 inhibitor has been developed as more efficient than cytostatic rapamycin. It suppresses cell proliferation by inhibiting the activity of both complexes - mTORC1 and mTORC2 [19, 29]. Indeed, it has been repeatedly shown that it is more effective in certain cell lines by blocking the cell cycle progression [24]. However, in ERas tumor cell line the anti-proliferative effect of pp242 is not due to the cell cycle arrest and inhibition of proliferation but rather a consequence of the activation of mitophagy followed by rapid cell death.

**Figure 10:**
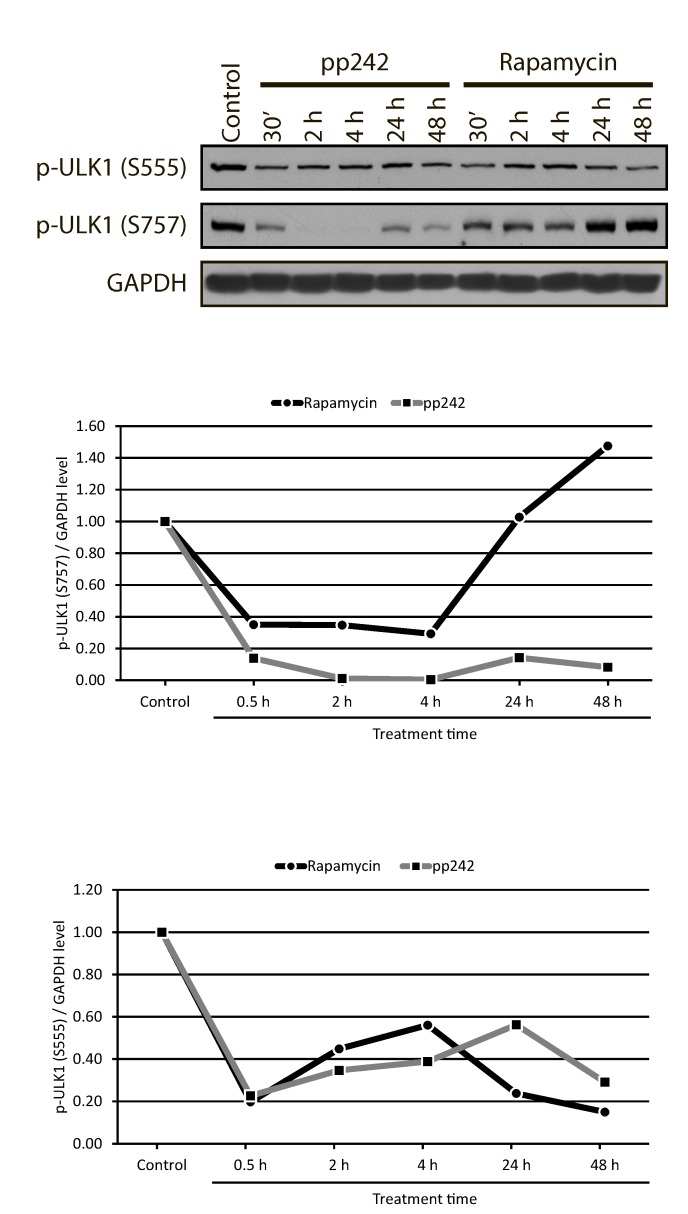
ULK1 phosphorylation at Ser757 and Ser 555 in mTOR inhibitor-treated ERas cells **A.** The rapid and complete inhibition of ULK1-Ser757 by the action pp242 is not restored even after 48 h, while rapamycin causes a reversible inhibition of ULK1-Ser757 phosphorylation. The level of ULK1-Ser555 phosphorylation changes in a similar way upon mTOR inhibition. Graphic representation of the blot data obtained after densitometry of ULK1-Ser757 **B.** and of ULK1-Ser555 **C.**

Almost complete inhibition of the mTORC1-4EBP1 pathway that leads to a decrease of 4EBP1 phosphorylation closely correlates with the inhibition of ULK1 phosphorylation at Ser757, which is necessary for the initiation of autophagy (Figure 10A). It is known that mTORC1 negatively regulates autophagy, and mTOR inhibitors can induce autophagy even on high level of amino acids [50]. The mTORC1 directly interacts with ULK1-ATG13-FIP200 complex and phosphorylates ULK1 at Ser757 that leads to the disruption of the interaction between the ULK1 and another activator of autophagy - AMPK [37, 51].

We found that the suppression of the mTORC1 activity by rapamycin and pp242 gives very different kinetics of ULK1-Ser757 phosphorylation, a marker of the beginning of the autophagic process. At the early stages, pp242 almost completely inhibited ULK1-Ser757 phosphorylation, whereas Rapa only slightly decreased its phosphorylation, but 24 h later, the level of ULK1 phosphorylation restored to a control value. Moreover, after 48 h it becomes even higher than in the control. In pp242-treated cells, phosphorylation of ULK1-Ser757 remains extremely low throughout the time of inhibitor treatment (Figure 10B). AMPK-dependent phosphorylation of ULK1 at Ser555 is capable of activating the process of autophagy in various cell lines. As expected, phosphorylation of ULK1-Ser555 decreases in the presence of both inhibitors in a similar manner, thus confirming the dominant role of mTORC1 in the regulation of autophagy in ERas cells (Figure 10C).

**Figure 11:**
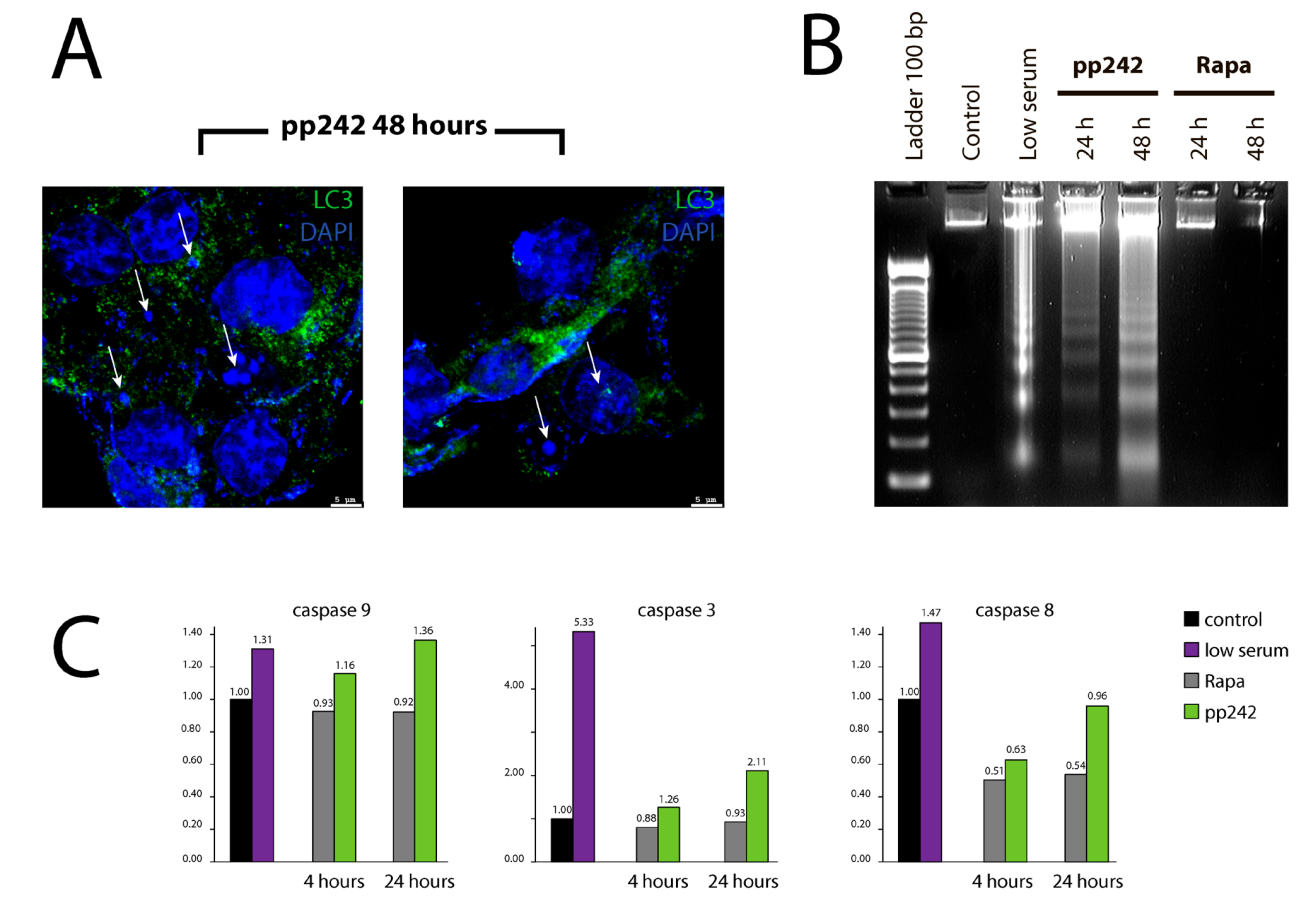
pp242-induced autophagy is terminated by apoptotic cell death type **A.** DAPI-positive material in the cytoplasm of pp242-treated cells expressing a marker of autophagy LC3. **B.** Nucleosomal DNA fragmentation in pp242-treated cells. **C.** Activation of caspases 3, 9 and 8 occurs only in pp242-treated cells.

According to our morphological data, pp242-induced mitophagy characterizes by the various destructive processes both in the cytoplasm and in the nuclei. A weak DAPI staining for DNA, loss of a clear outline of the nuclear envelope and the appearance of DAPI-positive material in the cytoplasm support this conclusion (Figure 11A). These events coincide in time with the flow cytometry data indicating an accumulation of a sub-diploid G1 peak (Figure 1E) that characterizes nuclear DNA degradation. According to morphology data, MTT test, and TEM analysis, the rapamycin-induced autophagy does not demonstrate the features of cell destruction neither in the cytoplasm nor in the nuclei.

According to the international nomenclature describing several types of cell death [31], the autophagic cell death type II is a caspase-independent. Analysis of caspase activity showed that pp242 treatment for 4 h and 24 h significantly increases the activity of caspase-9 and caspase-3 but to a lesser extent than serum deprivation (Figure 11C). The activity of caspase-8, which may enforce autophagic process and the formation of active caspase complexes on p62/SQSTM platform, did not markedly change after treatment with rapamycin and pp242. Rapamycin does not activate caspases 9, 3 and 8 types (Figure 11C). In addition to caspase assay, we assessed acidic phosphatase activity by using Gomori reaction to characterize the activity of lysosomes. By comparing the effects produced by rapamycin and pp242, one can see that in pp242-treated cells the acid phosphatase activity is much higher than in rapamycin-exposed cells (Supplementary Figure 1).

Despite the fact that flow cytometry and DAPI staining data reveal a DAPI-positive material in the cytoplasm of pp242-treated cells, no classical features of apoptotic cell death was identified: chromatin condensation, formation of apoptotic bodies or blebbing. However, DNA agarose gel electrophoresis showed that cells treated with pp242 contain DNA fragments characteristic for nucleosomal-like DNA degradation. In contrast, rapamycin does not induce similar DNA fragmentation (Figure 11B). Thus, the final step of pp242-induced mTOR-dependent autophagy is the cell death accompanied by apoptosis-like DNA degradation.

**Figure 12:**
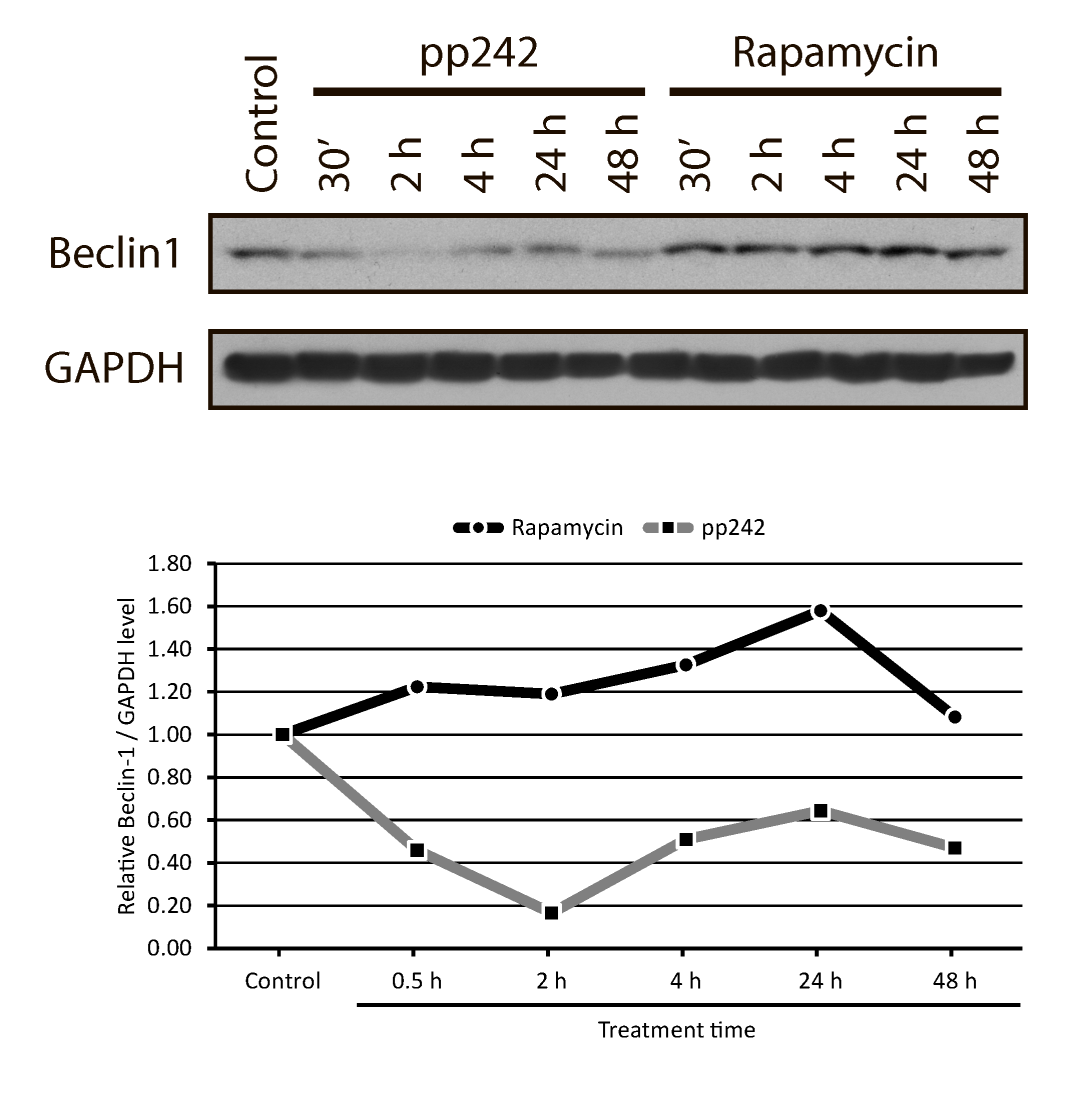
Western blot analysis of the Beclin1 content in ERas cells treated with rapamycin and pp242 Pp242 decreases the content of Beclin1, while rapamycin causes its accumulation, below -graphic representation of the blot data obtained after densitometry of Beclin1.

To activate autophagy, ULK1-ATG13-FIP200 complex should be dissociated from a negative regulator of autophagy - VPS34-Beclin 1 complex, which is normally inactivated by binding to anti-apoptotic protein Bcl2 [52]. When Beclin 1 is active, the process of nucleation takes place thereby giving rise to further development of the autophagic process. In ERas cells treated with pp242 the Beclin1 content greatly reduced after 30 min and 2 h treatment, whereas rapamycin causes its accumulation (Figure 12) that is an agreement with the results characterizing the LC3-I in LC3-II conversion (Figure 6). It seems that apoptosis-induced cas­pase activation can promote degradation of a number of essential autophagy proteins, slowing down the autophagic process and converting pro-autophagic proteins into pro-apoptotic derivatives.

## DISCUSSION

There are many evidences that mTOR signaling pathway controls the processes of proliferation, cell growth, cell death and aging [2, 53–55]. By stimulating the anabolic processes, mTOR inhibits autophagy, which is the main catabolic process of cells involved in modulation of cell death and aging. In particular, mTORC1 inhibits the formation of autophagosomes by phosphorylation of pro-autophagic kinase ULK1 thereby preventing its activation by AMPK kinase. Besides, mTORC1 also phosphorylates and inhibits ATG13 - a positive regulator of ULK1 [13–15]. Furthermore, mTORC1 inhibits autophagy by blocking the biogenesis of lysosomes by inhibiting phosphorylation and nuclear translocation of transcription factor EB (TFEB) [13]. Also, mTORC1 can control both microautophagy designed for disposal of defective cytosolic proteins and macroautophagy - a process of destruction of damaged organelles. This type of autophagy has protective properties elaborated for saving the cells from death associated with massive damages.

In this work, we studied whether mTORC1 inhibitor rapamycin and mTORC1/mTORC2 inhibitor pp242 are able to suppress proliferation and induce autophagic death in ERas transformed cells. These cells are resistant to rapamycin, but pp242 effectively kills them. Correspondingly, these inhibitors differ in their ability to cause different forms of autophagy: Rapa induces a non-selective autophagy, whereas pp242 triggers massive selective mitophagy terminated by cell death. This correlates with the different kinetics of mTORC1-4EBP1 inhibition. If pp242 completely inhibits phosphorylation of 4EBP1 in the first hours of treatment, the effect of rapamycin occurs only after 24 h-treatment, when according to the expression of markers autophagy decreases.

Similar data indicating on tight connection between the inhibition of mTORC1-4EBP1 pathway and suppression of proliferation have been before obtained [19, 22, 26–28]. Other results indicate that pp242 suppresses more effectively mTORC1-S6K-S6 pathway [19, 56, 57]. It is still unclear until now why Rapa and pp242 have different efficiency of suppression of the mTORC1 downstream targets and how it is related to their anti-proliferative effects.

Some authors believe that mTOR kinase inhibitors like pp242 are more effective as compared to Rapa due to their ability to inhibit both complexes - mTORC1 and mTORC2 [18, 20]. However, other studies have shown that even in mTORC2-deficient cells the mTOR kinase inhibitors suppresses cell proliferation more effectively than Rapa [19, 24]. This suggests that the ATP-competitive kinase inhibitors are able to overcome the rapamycin-resistance of mTORC1 in some types of tumor cells. In addition, although previously thought that Rapa inhibits only mTORC1 complex but mTORC2 is insensitive to it, the subsequent data showed that a longer rapamycin treatment effectively inhibits the mTORC2 activity too [58]. In our case, however, the kinetics of mTORC2 suppression assessed by PKB/Akt-Ser473 phosphorylation is similar upon treatment with rapamycin and pp242 that does not allow explaining distinctions in their anti-proliferative effect.

Currently, it is unclear what can be used as a criterion for the anti-proliferative effectiveness of the rapamycin and rapalogs and the inhibitors of mTOR kinase domain - the results of inhibition of mTORC1-S6K1-S6 or mTORC1-4EBP1 axes, as available data are controversial. In our study, the kinetics of suppression of mTORC1-pS6 by both inhibitors is very similar with the maximum effect on 24 h of treatment. Despite the role of S6K1 and S6 protein in regulation of cell proliferation, the *s6k1* knockout mice do not demonstrate a decrease of protein synthesis in the liver and muscles as compared with the control mice [59, 60]. However, if Rapa decreases the protein content per cell that leads to a reduction of ERas cell size, the pp242 does insignificantly affect the protein content and cell size. Given that *S6k1^−/−^ S6k2^−/−^* cells did not show a decrease of the translation level even though having a defective ribosome biogenesis, some authors believe that there is a compensatory mechanism of protein synthesis still not exactly identified [59]. Furthermore, kinase inhibitor Torin1 has only a minor effect on global translation in *4EBP1*-deficient cells. These data suggest that mTORC1 controls the global translation machinery mainly through 4EBP1 [61].

Activity of mTORC1-S6K-S6 pathway seems not to determine the suppression of proliferation of ERas cells treated for 24 h and 48 h with Rapa and pp242. At these time-points, the cells treated with pp242 undergo a massive death, while Rapa -treated cells rapidly proliferate, retaining similar levels of pS6. Rapa -treated cells undergo a short suppression of proliferation, and then, in parallel with the activation of non-selective autophagy, restore proliferation. Thus, Rapa-induced autophagy has a cytoprotective character, moreover, the autophagy acts as a factor that retain the proliferative potential of the tumor cells. Such an approach has been described for stem cells, which after rapamycin treatment can acquire additional “rejuvenation” and should proliferate more vigorously [62]. Recently, Leontieva et al. showed that at low doses (10-30 nM) pp242 and Torin1 suppress geroconversion in p21-arrested human cells [63]. Thus inhibiting proliferation by then selves, mTOR inhibitors can maintain re-proliferative potential and small-cell morphology.

It is now established that the high level of mTORC1-4EBP1 activity is characteristic for a number of tumors [64]. Importantly, ERas cells have a very high level of mTORC1-4EBP1 activity. One of the mechanisms of resistance of tumor cells to drugs targeting the mTORC1 can be based on the sustained activity of mTORC1-4EBP1/eIF4E pathway [65–67].

We first found that the mechanism of anti-proliferative effect of pp242 is associated with the suppression of mTORC1-4EBP1 and activation of mitophagy followed by cell death. This process is time-coordinated with the suppression of ULK1,2 phosphorylation at Ser757. Rapa is less effective in suppression of mTORC1- 4EBP, it transiently suppresses phosphorylation of ULK1-Ser757 and is not capable of inducing the mitophagy. This is a mechanistic explanation of a resistance of ERas transformed cells to rapamycin. Very high, non-physiological concentrations of rapamycin (20 000 nM) can cause apoptotic-like cell death in human tumor cell line MDA-MB231, but can't in MCF-7 [68]. The authors did not show whether this effect is caused by mitochondrial damage similar to that in our work, although they observed a correlation with the inhibition of 4EBP1 phosphorylation. However, we were unable to induce the cell death, even using the same concentrations of Rapa (20 000 nM). Instead, there was a temporary suppression of cell proliferation followed by restoration of proliferation according clonogenic survival data.

Currently, the mitochondria are the key organelle, which is targeted by the agents that induce mitochondria-dependent apoptosis or so-called «intrinsic pathway of apoptosis» [69]. The main factor for inducing apoptosis through a mitochondrial pathway is mitochondrial outer membrane permeabilisation (MOMP) leading to a release of cyt C inside the cytosol and the caspase activation. According to IF microscopy, pp242 increases MOMP within 2,5- 4 h, activates mitophagy and accumulation of cyt C in the autophagic vacuoles as assessed by IF and TEM. After 24 h, virtually all cyt C detects in autophagolysosomes that correlates with apoptotic DNA fragmentation. In cells treated by pp242 for 48 h, cyt C is completely destroyed in autophagolysosomes, and DNA fragmentation process is even more intense. Lesion of MOMP causes a release of not only cyt C, but also catabolic hydrolases and activators of caspases that alter the membrane potential resulting in an energy catastrophe and cell death. We believe that in ERas transformed cells pp242-induced death might be classified as a mixed type of death - autophagy + apoptosis [70].

Autophagy and apoptosis have common links in the signaling pathways and complement each other [70]. It is shown that mitophagy is one of the mechanisms by which a cell can reduce the level of apoptosis [71]. In contrast to rapamycin, which does not cause mitophagy, in pp242-treated cells the destruction of the damaged mitochondria continues throughout the inhibitor presence. Autophagic removal of the damaged mitochondria does not prevent activation of apoptosis and cell death. Moreover, apoptosis may be an additional factor contributing to the degradation of proteins involved in the regulation of autophagy thereby enhancing cell death. Since the content of Beclin 1, a key regulator of autophagy [72, 73], is greatly reduced in pp242-treated cells, this dysregulates the coordination of different stages of autophagy. Recent data show that AMBRA1, an autophagy activator (Activating Molecule in BECN1 Regulated Autophagy 1) can be irreversibly destroyed by calpain- and caspase-dependent pathways [74]. Correspondingly, cells with mutations in the AMBRA1 sites cleaved by caspase-3 are resistant to induction of apoptosis program [75]. In addition, autophagy can activate apoptosis *via* the caspase-mediated cleavage of apoptosis inhibitors IAP [76].

Comparing the effects of Rapa and pp242, we found that even extremely high doses of rapamycin are not able to induce cell death in tumor ERas cells. Recent data published advance have demonstrated that those human cell lines that have the c*Ha-ras* mutations (but not mutations of Ki- or N-Ras) will die by apoptosis upon treatment with mTOR kinase inhibitors and inhibitors of MEK (doi: 10.18632/oncotarget.5619). Our data suggest that the mTOR kinase inhibitors can be considered as very promising anticancer agents for the Ras-expressing cells due to activation of mitophagy followed by apoptotic cell death.

## MATERIALS AND METHODS

### Cell culture and treatment

Cells with stable expression of adenoviral E1Aas5 and cHa-ras proteins were selected from rat embryonic fibroblasts co-transfected with E1A- region of Ad5 viral DNA and human cHa-Ras gene with amino acid substitution at position 12 and 61. Cells were cultured in DMEM supplemented with 10% fetal calf serum (FCS), penicillin, and streptomycin in 5% CO2 at 37°C.

### Antibodies

#### Primary antibodies

Phospho-S6 Ribosomal Protein (Ser235/236) - #2211,

Phospho-4E-BP1 (Thr37/46) - #9459,

Beclin-1 - #3738

Phospho-Akt (Ser473) - #4060,

GAPDH - #2118,

LAMP1 - #9091,

Phospho-ULK1 (Ser757) - #6888,

Cytochrome c - #4272 all by Cell Signaling Technology;

Anti-phospho-ULK1 (Ser555) Antibody - 2041599 by EMD Millipore;

Anti-p62 Ick ligand - 610832 by BD Biosciences;

Anti-LC3 - PM036 by MBL.

#### Secondary antibodies

Goat anti-Rabbit IgG (H+L) Secondary Antibody, Alexa Fluor^®^ 488 conjugate - A11008,

Goat anti-Mouse IgG (H+L) Secondary Antibody, Alexa Fluor^®^ 568 conjugate - A11031 all by Invitrogen;

Anti-Rabbit IgG, HRP conjugate - A0545,

Anti-Mouse IgG, HRP conjugate - A9044 all by Sigma-Aldrich.

### Flow cytometry

To assay cell cycle distribution cells flow cytometry assay of propidium iodide-stained cells was performed as described before [77].

### Growth curves

Cells were seeded at the initial density of 3×10^4^ cells per 30-mm dish in 3 repeats 24 h before the treatment. Cells were treated or left untreated and counted in cell counting chamber every day up to 5-7 days. The medium was replaced by the fresh one supplemented with 10% FCS every second day. The growth curve was made based on the data obtained in 3 independent experiments.

### Morphological staining

with Eosine metylene blue according to protocol May-Grunwald.

To analyze morphology of ERas cells were grown on coverslips, washing in PBS and stained 45 sec, as previously described [77].

### MTT viability assay

For experiments, the cells were plated on 96-well plates at the initial density of 2500 cells per well in triplicates. After 24 h of growth, the cells were treated with drugs as indicated in figures. For the test, the cells were incubated in the conditioned medium with 0.5 mg/ml MTT (Sigma-Aldrich) for 2 h at 37°C in a CO_2_-incubator. The medium was changed to DMSO, and the optical density was measured at 570 nm using DMSO as a blank solution. The optical densities were normalized to those of untreated cells in 24 h after plating.

### Clonogenic survival

The ability of cells to proliferate at the clonal density after rapamycin treatment was studied by the method of clonogenic survival. After 48 h rapamycin treatment the cells were plated at the density of 200 cells per 30 mm dish in triplicates. After 5d clones were fixed and stained with a solution containing 10% acetic acid, 30% ethanol, and 0.25% brilliant blue. The number of grown up clones was counted, and dishes were photographed.

### Immunoblotting

Cells were lysed in a buffer containing 10 mM Tris-HCl pH 7.4, 150 mM NaCl, 50 mM NaF, 1% NP-40, 0.5% SDS containing protease inhibitors cocktail (Roche), 1 mM Na_3_VO_4_, 1 mM phenylmethylsulfonyl fluoride, and 1 mM dithiothreitol. Thirty micrograms of total protein were separated by SDS-PAGE and electro-blotted onto nitrocellulose (0.45μm, Bio-Rad) or PVDF (0.2 μm, EMD Millipore) membrane. The membranes were blocked in 5% skimmed milk (Santa-Cruz) and probed with primary antibodies. The blots were washed, incubated with secondary horseradish peroxidase-conjugated secondary antibodies and developed by enhanced chemiluminescence (ECL, ThermoFisher Scientific). Western blot densitometry was performed using Gel-Pro Analyzer 3.0 software (Media Cybernetics). The optical densities were normalized to those of control samples.

### Immunofluorescence and confocal microscopy

For immunofluorescence analysis, cells grown on coverslips were fixed with 3.7% paraformaldehyde in PBS for 15 min. Cells were washed with PBS and permerabilized with 0.1% Triton X-100 in PBS for 30 min followed by incubation in blocking solution (5% goat serum in PBS containing 0.5% Tween-20 (PBST)) for 1 h. Cells were incubated with primary antibodies diluted in blocking solution overnight at 4°C or rhodamine phalloidin (R415, Invitrogen) 10 min at 37°C for actin staining, washed with PBST, and incubated with secondary antibodies Alexa-488 and Alexa- 568 (Invitrogen) for 1 h at room temperature. Coverslips were mounted using ProLong Gold (P36931, Invitrogen) mounting medium containing 4,6-diamidino-2-phenylindole (DAPI). Cells were analyzed with Leica TCP SP5 scanning confocal microscope (Leica Microsystems). Confocal images were acquired using a Leica TCS SP5 microscope (Leica Microsystems) and LAS AF software (Leica Microsystems).

### Caspase activity assay

Evaluation of caspase-3, 8 and 9 activity was conducted using fluorogenic substrates: for caspase-3 - Ac-DEVD-AMC (EMD Millipore), for caspase-8 - Ac-VETD-AMC (Sigma-Aldrich) and caspase-9 - Ac-LEHD-AFC (EMD Millipore). Cells were lysed in a buffer containing 10 mM HEPES pH 7.4, 0.1% CHAPS, 0.5% IGEPAL, 5 mM DTT for 30 min at 4° C. The reaction mixture (500 μl) consisted of 50 μg of total protein lysate in reaction buffer (10 mM HEPES pH 7.4, 0.1% CHAPS, 1 mM DTT) containing 40 μM fluorogenic substrate. The reaction was conducted for 1 h at 37° C in the dark. The emission fluorescence at 460 nm produced by cleaved Ac-DEVD-AMC was measured after excitation at 360 nm using fluorimeter GloMax^®^-Multi Jr. Data are presented as mean ±S.E.M. of three independent replicates (*n* = 3). The values were normalized to those of control samples.

### DNA isolation and electrophoresis

To analyze oligonucleosomal DNA fragmentation, cells were lysed in a buffer containing 5 mM Tris-HCl, 0.5 % Triton X-100 and 100 mM EDTA pH 8.0 for 20 min at 4^0^C. Lysates were centrifuged at 12000 g, then NaCL and RNAse A were added to the supernatant (the final concentration - 0,15 M and 100 μg/ml). The probes were incubated for 1 h at 37°C. Then probes were supplemented with SDS and proteinase K (the final concentration - 0.5 % и 200 g/ml) and incubated for 2 h at 37°C. DNA was deproteinized by a mixture of water-saturated phenol pH 8.0/chloroform. DNA was re-precipitated, washed with 70% of ethanol, and dried. For electrophoresis, DNA was dissolved in 1 mM TE buffer pH 8.0 and subjected to electrophoresis in 2% agarose gel (Sigma). Gel was stained with ethidium bromide to visualize DNA fragmentation bands.

## SUPPLEMENTARY MATERIAL FIGURE



## Abbreviations

qRT-PCR: quantitative RT-PCR
MTT: (3-(4,5-Dimethylthiazol-2-yl)-2,5-diphenyltetrazolium bromide
PBS: phosphate saline solution
BSA: bovine serum albumin
DAPI: 4,6-diamidino-2-phenylindole
SDS: sodium dodecyl sulphate
DMSO: dimethyl sulfoxide
FCS: fetal calf serum
DMEM: Dulbecco's modified Eagle medium
mTOR: mammalian target of rapamycin
CHAPS: 3-[(3-Cholamidopropyl)dimethylammonio]-1-propanesulfonate hydrate
TEM: transmission electron microscopy
