# Supplementary material for: mTOR kinase inhibitor pp242 causes mitophagy terminated by apoptotic cell death in E1A-Ras transformed cells
Source: Oncotarget. 2015 Dec 4;6(42):44905–26. doi: 10.18632/oncotarget.6457 (PMC4792600; doi:10.18632/oncotarget.6457)
Supplement: Supplementary file 1 [file oncotarget-06-44905-s001.pdf]

mTOR kinase inhibitor pp242 causes mitophagy terminated by apoptotic cell death in E1A-Ras transformed cells

Supplementary Material

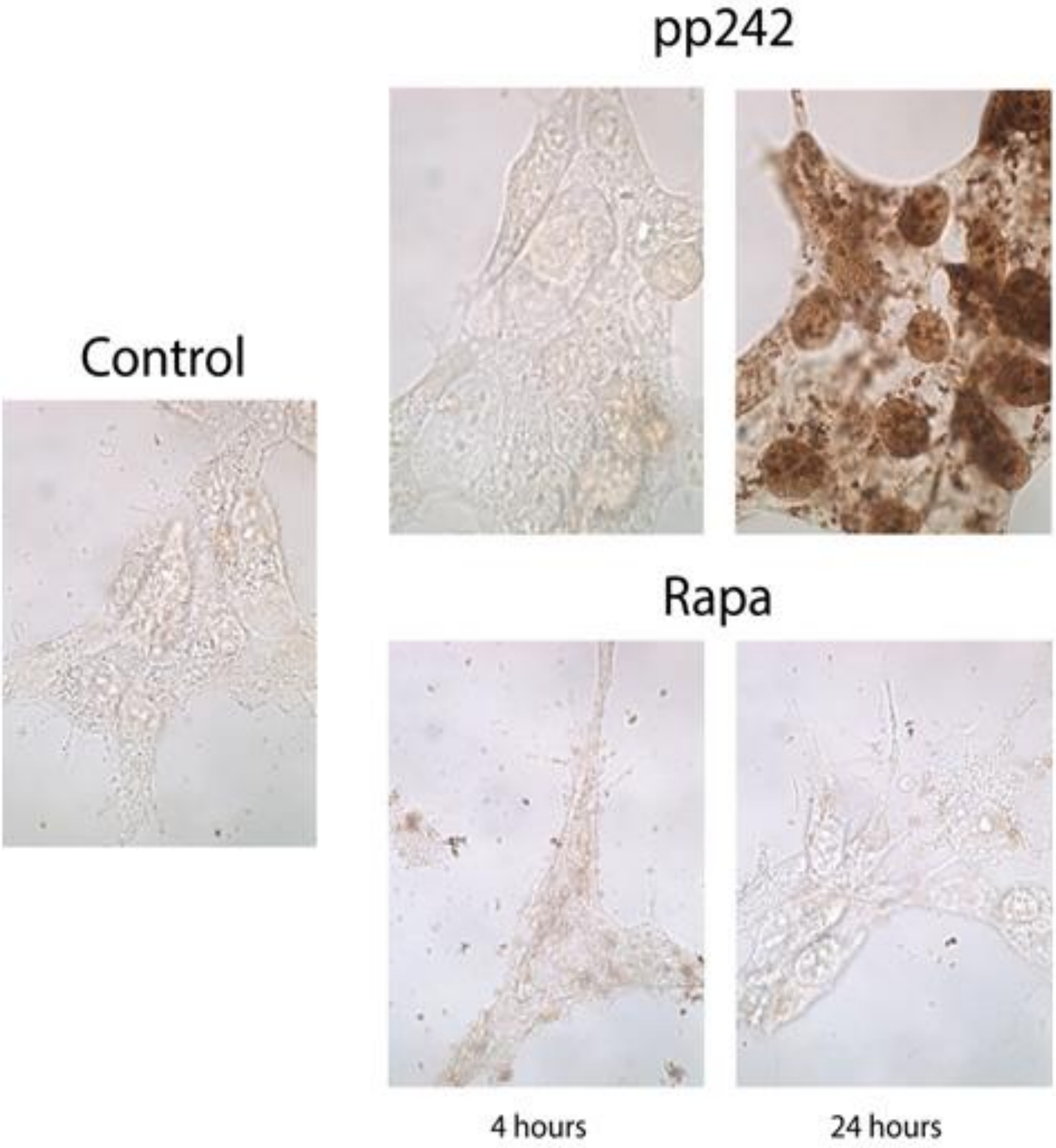

Supplementary Figure 1. Acid phosphatase activity assessed by Gomory staining, a marker of lysosomal activity, in control and treated with rapamycin or pp242 ERas . Cells were fixed on the coverslip in 4% formaldehyde for 1 h at room temperature and then incubated in the freshly prepared and filtered glycerophosphate medium at 37°C for 1 h. The medium consisted of 0.01 M Na-ß-glycerophosphate, 0.05 M sodium acetate buffer (pH 5.0), and 0.004 M Pb(NO3)2. Coverslips were then rinsed with distilled water, immersed in 1% solution of ammonium sulfide for 1-2 min, washed again with running water, mounted on glass slides, and examined in a laser scanning confocal microscope Leica TCS SPS (Leica Microsystems, Bucks, UK) in the reflected light (wavelength 543 nm).
